# Osteocalcin promotes bone mineralization but is not a hormone

**DOI:** 10.1371/journal.pgen.1008714

**Published:** 2020-06-02

**Authors:** Stavros C. Manolagas

**Affiliations:** 1 Division of Endocrinology and Metabolism, Center for Osteoporosis and Metabolic Bone Diseases, University of Arkansas for Medical Sciences, Arkansas, United States of America; 2 Central Arkansas Veterans Healthcare System, Little Rock, Arkansas, United States of America; Murdoch Childrens Research Institute, AUSTRALIA

During the last 24 years the bone and mineral metabolism research community (and the National Institutes of Health and numerous other national and international funding agencies that support it) have devoted a great deal of intellectual energy and resources to some provocative, sometimes controversial, ideas about osteocalcin (OCN). OCN is a 46 amino-acid protein that is produced and secreted almost exclusively by osteoblasts, terminally differentiated cells responsible for the synthesis and mineralization of bone matrix during development of the skeleton and its periodic regeneration throughout life. Osteoblasts originate from mesenchymal progenitors and are short-lived cells that are constantly replaced, depending on the demand for bone formation in a particular location and time [1]. OCN secreted by osteoblasts contains three γ-carboxyglutamic acid residues that confer high affinity to the bone hydroxyapatite matrix. However, when bone is resorbed by osteoclasts, a macrophage-derived cell type, the acidic pH in the resorption compartment causes the carboxyl groups on OCN to be removed, and decarboxylated OCN is released into circulation. The circulating levels of decarboxylated OCN are, therefore, dependent on the rate of bone turnover, also known as remodeling.

Originally thought to function exclusively in bone, a more expansive view of decarboxylated OCN as an endocrine hormone has evolved, largely through work of Gerard Karsenty and colleagues and beginning with the description of an OCN knockout mouse 24 years ago [2]. As a hormone, OCN has been proposed to act on multiple end organs and tissues including the pancreas, liver, fat cells, muscle, male gonads, and brain to regulate functions ranging from bone mass accumulation to body weight, adiposity, glucose and energy metabolism, male fertility, brain development, and cognition [2–6]. This idea—that OCN is an endocrine hormone with pleiotropic effects—is widely cited in textbooks and review articles [7,8] and has provided the rationale for numerous human studies on the relationship between OCN and diabetes or obesity.

There have been, however, several apparent shortcomings in the “OCN is an endocrine hormone” idea. The number of osteoblasts (and therefore the circulating levels of decarboxylated OCN) inexorably change throughout life as a result of physiologic, adaptive, or pathologic changes of bone itself that can be acute or chronic, systemic or localized, and reversible or irreversible, without changes in the putative extraskeletal targets of decarboxylated OCN. Examples are skeletal development, growth, adaptation of the skeleton to mechanical forces, fracture healing, changing calcium needs, stress, menstrual cycle, pregnancy, lactation, menopause, aging, hyperparathyroidism or hypoparathyroidism, hyperthyroidism, hypercortisolemia, Paget’s disease, bone tumors, etc. Similarly, medications—approved after extensive trials with thousands of subjects and subsequently used by millions for the treatment of osteoporosis—dramatically decrease or increase serum OCN levels without any effect on glucose homeostasis, testosterone production, muscles, or behavior. In addition, mouse gene targeting studies of GPRC6A, proposed to be an OCN receptor that modulates pancreatic β-cell proliferation [3], have yielded conflicting results with regard to glucose and energy metabolism [9–11]. One possible explanation for the differences between results in mice and those in humans is that OCN genetics and function differ between humans and mice; humans have a single OCN gene, whereas in mice, there are two adjacent OCN genes, Bglap and Bglap2. However, rats have a single OCN gene, and rats carrying an OCN null mutation introduced by gene editing do not exhibit obesity, insulin resistance, or glucose intolerance [12]. Sorting through these apparent discrepancies has been challenging since the Karsenty knockout mouse has not been widely available for confirmatory studies.

The current issue of *PLOS Genetics* features two studies of independent OCN mouse knockout models. In the article by Moriishi and colleagues [13], the authors replaced DNA encoding Bglap and Bglap2 with a neo cassette in embryonic stem cells. Using this model, they investigated the role of OCN on bone formation and mineralization, as well as glucose metabolism, testosterone production, and muscle mass. They show that, in contrast to the results reported by Karsenty and colleagues (which used a similar gene targeting strategy), OCN plays no role in bone formation (or resorption) and bone mass in the estrogen-sufficient or estrogen-deficient state. Instead, OCN is indispensable for the alignment of biological apatite crystallites parallel to collagen fibers (Fig 1). Loss of OCN function had no effect on collagen orientation, which remained normal. Bone strength, however, was decreased in the OCN-deficient mice indicating that alignment of crystallites with collagen fibers is one of the elusive determinants of bone quality that together with bone mass determines the ability of bone to resist fractures. Additionally, the detailed and thoughtful study by Moriishi and colleagues shows that OCN plays no role in exercise-induced bone formation, glucose metabolism, improvement of glucose metabolism caused by exercise, testosterone synthesis, spermatogenesis, or muscle mass.

**Fig 1:**
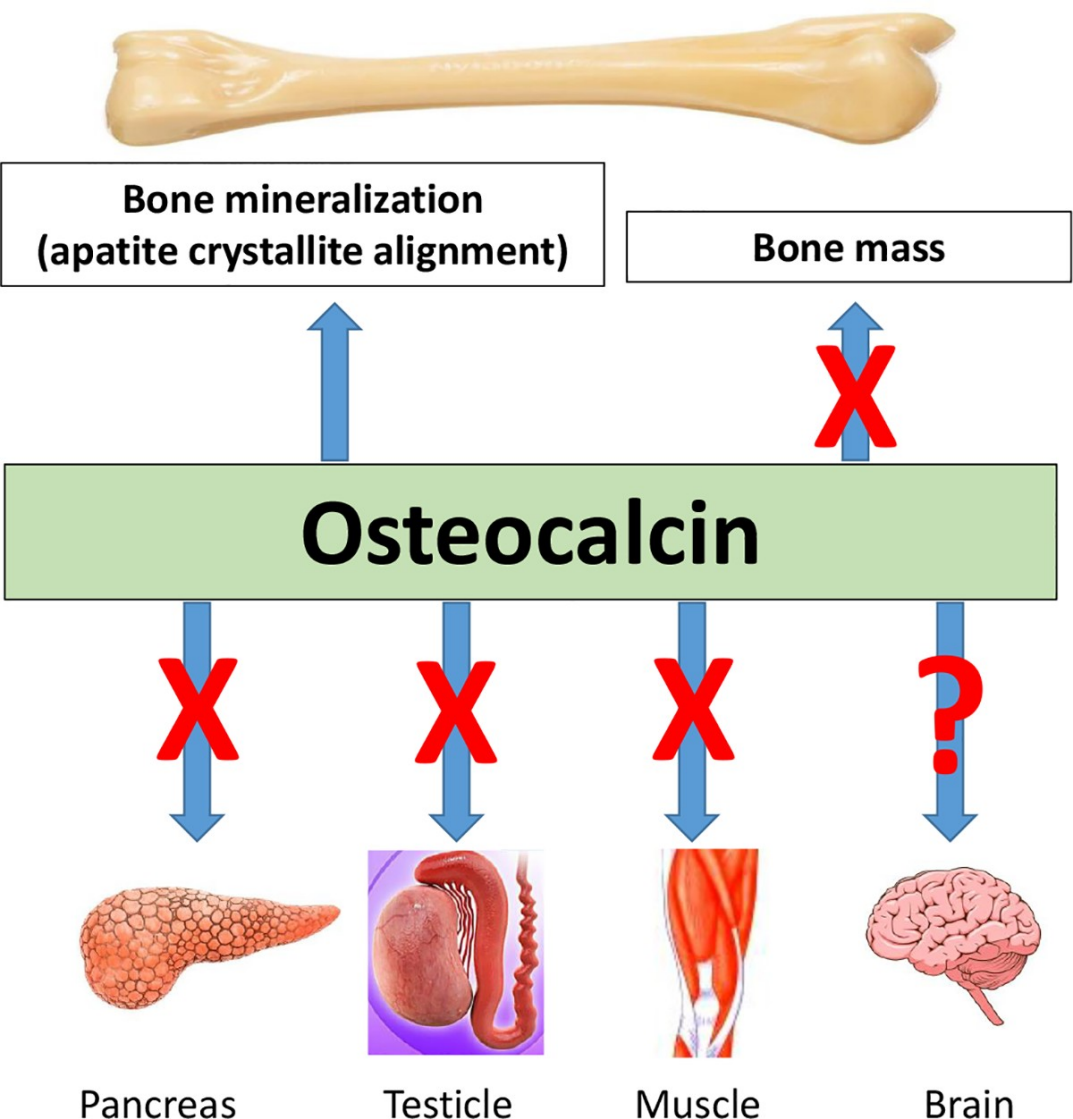
A 2020 update of the biologic role of osteocalcin.

In the article by Diegel and colleagues [14], the authors used CRISPR/Cas9-mediated gene editing to delete most of the Bglap and Bglap2 protein coding regions. They report that homozygous gene-edited mice have no circulating OCN but normal bone mass as well as normal blood glucose and normal male fertility. Additionally, RNA-seq of cortical bone samples from the OCN-deficient mice show minimal differences from the nonmutant control mice. The mutant mice do, nonetheless, exhibit increased bone crystal size and maturation of hydroxyapatite, consistent with the aforementioned report of Moriishi and colleagues, earlier evidence by many other groups, and the general consensus that OCN plays a role in mineralization.

What are we to make of the apparent discrepancies between the articles in the current issue of *PLOS Genetics*, the work on the rat knockout [12], and the previous body of work by Karsenty and colleagues? In the last few years, irreproducibility of research results, particularly from studies with preclinical animal models that could be relevant to human biology and disease, has become an enormous concern for all stakeholders of the research enterprise [15], including the bone and mineral research community [16]. The studies discussed in this perspective are a prime example of the problem. Genetic background, modifier genes, and differences in the molecular genetics of the knockout alleles remain possible explanations for the discrepancies, but neither Moriishi and colleagues nor Diegel and colleagues can explain the striking incongruence between their findings and those from the Karsenty group. Importantly, however, both groups explicitly state that the animals they have constructed will be donated to distribution centers and made publicly available so that their findings can be confirmed and extended by other interested investigators. Indeed, the importance of resource sharing is one of the most valuable take-home messages. I believe that science is inexorably self-correcting, and the work in the current issue of *PLOS Genetics* represents an important correction with implications for past and future work on the connections, or lack thereof, between bone and the rest of the body.

## References

[1] Manolagas SC. (2000) Birth and death of bone cells: basic regulatory mechanisms and implications for the pathogenesis and treatment of osteoporosis. Endocr Rev. 21:115–37. 10.1210/edrv.21.2.0395 10782361

[2] Ducy P, Desbois C, Boyce B, Pinero G, Story B, et al (1996) Increased bone formation in osteocalcin-deficient mice. Nature. 1:448–52. 10.1038/382448a0 8684484

[3] Lee NK, Sowa H, Hinoi E, Ferron M, Ahn JD, et al (2007) Endocrine regulation of energy metabolism by the skeleton. Cell 130:456–469. 10.1016/j.cell.2007.05.047 17693256 PMC2013746

[4] Oury F, Sumara G, Sumara O, Ferron M, Chang H, et al (2011) Endocrine regulation of male fertility by the skeleton. Cell. 144:796–809. 10.1016/j.cell.2011.02.004 21333348 PMC3052787

[5] Oury F, Khrimian L, Denny CA, Gardin A, Chamouni A, et al (2013) Maternal and offspring pools of osteocalcin influence brain development and functions. Cell. 26:228–41. 10.1016/j.cell.2013.08.042 PMC3864001 24074871

[6] Mera P, Laue K, Wei J, Berger JM, Karsenty G. (2016) Osteocalcin is necessary and sufficient to maintain muscle mass in older mice. Mol Metab. 16:1042–1047. 10.1016/j.molmet.2016.07.002 PMC5034485 27689017

[7] Karsenty G. (2017) Update on the biology of osteocalcin. Endocr Pract. 23:1270–1274. 10.4158/EP171966.RA 28704102

[8] Ferron M and Karsenty G. Regulation of energy metabolism by bone-derived hormones In *Principles of Bone Biology*. Bilezikian JP, Martin TJ, Clemens TL, Rosen CJ (eds). Academic Press 2020.

[9] Wellendorph P, Johansen LD, Jensen AA, Casanova E, Gassmann M, et al No evidence for a bone phenotype in GPRC6A knockout mice under normal physiological conditions. (2009) J Mol Endocrinol. 42: 215–223. 10.1677/JME-08-0149 19103720

[10] Jørgensen CV, Gasparini SJ, Tu J, Zhou H, Seibel MJ, et al (2019) Metabolic and skeletal homeostasis are maintained in full locus GPRC6A knockout mice. Scientific reports. 9:5995 10.1038/s41598-019-41921-8 30979912 PMC6461682

[11] Smajilovic S, Clemmensen C, Johansen LD, Wellendorph P, Holst JJ, et al (2013) The L-alpha-amino acid receptor GPRC6A is expressed in the islets of Langerhans but is not involved in L-arginine-induced insulin release. Amino acids. 44:383–390. 10.1007/s00726-012-1341-8 22714012

[12] Lambert LJ, Challa AK, Niu A, Zhou L, Tucholski J, et al (2016) Increased trabecular bone and improved biomechanics in an osteocalcin-null rat model created by CRISPR/Cas9 technology. Dis Model Mech. 9:1169–1179. 10.1242/dmm.025247 27483347 PMC5087831

[13] Moriishi T, Ozasa R, Ishimoto T, Nakano T, Hasegawa T, et al (2020) Osteocalcin is necessary for the alignment of apatite crystallites, but not glucose metabolism, testosterone synthesis, or muscle mass. PLoS Genetics. 16:e1008586 10.1371/journal.pgen.1008586 32463816 PMC7255595

[14] Diegel CR, Hann S, Ayturk U, Hu JCW, Lim K, et al (2020) An osteocalcin-deficient mouse strain without endocrine abnormalities. PLoS Genetics. 16:e1008361 10.1371/journal.pgen.1008361 32463812 PMC7255615

[15] National Academies of Sciences, Engineering, and Medicine, Policy and Global Affairs, Committee on Science, Engineering, Medicine, and Public Policy, Committee on Responsible Science (2017) Fostering Integrity in Research. Washington (DC): National Academies Press (US); 4 11 10.17226/21896 . 29341557

[16] Manolagas SC, Kronenberg HM. (2014) Reproducibility of results in preclinical studies: a perspective from the bone field. J Bone Miner Res. 29:2131–40. 10.1002/jbmr.2293 24916175 PMC4356005

